# Low Vitamin K Status and Risk of Chronic Obstructive Pulmonary Disease

**DOI:** 10.3390/biomedicines13040807

**Published:** 2025-03-27

**Authors:** Daniel Alexander Ackermann, Allan Linneberg, Ema Rastoder, Anna Kubel Vognsen, Anne Ahrendt Bjerregaard, Lennart Friis-Hansen, Niklas Rye Jørgensen, Caroline Emma Hedsund, Niklas Dyrby Johansen, Daniel Modin, Maria Dons, Mats C. Højbjerg Lassen, Kristoffer Grundtvig Skaarup, Ditte Vesterlev, Mia Moberg, Julie Janner, Josefin Eklöf, Lars Pedersen, Elisabeth Bendstrup, Christian B. Laursen, Jørn Carlsen, Tor Biering-Sørensen, Jens-Ulrik Stæhr Jensen, Pradeesh Sivapalan

**Affiliations:** 1 Respiratory Medicine Section, Department of Medicine, Copenhagen University Hospital—Herlev and Gentofte, 2730 Copenhagen, Denmark; 2 Center for Clinical Research and Prevention, Copenhagen University Hospital-Bispebjerg and Frederiksberg, 2000 Copenhagen, Denmark; 3 Department of Clinical Medicine, Faculty of Health and Medical Sciences, University of Copenhagen, 2200 Copenhagen, Denmark; 4 Department of Clinical Research, University of Southern Denmark, 5000 Odense, Denmark; 5 Department of Clinical Biochemistry, Copenhagen University Hospital Rigshospitalet, 2100 Copenhagen, Denmark; 6 Department of Cardiology, Copenhagen University Hospital—Herlev and Gentofte, 2900 Hellerup, Copenhagen, Denmark; 7 Center for Translational Cardiology and Pragmatic Randomized Trials, Department of Biomedical Sciences, Faculty of Health and Medical Sciences, University of Copenhagen, 2200 Copenhagen, Denmark; 8 Department of Respiratory Medicine, Amager-Hvidovre Hospital, 2650 Copenhagen, Denmark; 9 Department of Respiratory Medicine and Infectious Diseases, Copenhagen University Hospital Bispebjerg, 2400 Copenhagen, Denmark; 10 Department of Respiratory Diseases and Allergy, Aarhus University Hospital, 8200 Aarhus, Denmark; 11 Department of Clinical Medicine, Aarhus University, 8000 Aarhus, Denmark; 12 Department of Respiratory Medicine, Odense University Hospital, 5000 Odense, Denmark; 13 Odense Respiratory Research Unit (ODIN), Department of Clinical Research, University of Southern Denmark, 5000 Odense, Denmark; 14 Department of Cardiology, Rigshospitalet, Copenhagen University Hospital, 2100 Copenhagen, Denmark; 15 Steno Diabetes Center Copenhagen, 2100 Copenhagen, Denmark

**Keywords:** vitamin K, COPD, dp-ucMGP, biomarker

## Abstract

**Background:** Vitamin K is a cofactor necessary for the biological activity of proteins like Matrix Gla Protein (MGP), which reduce calcification and help preserve lung function. This study aims to determine, first, whether low vitamin K status is associated with chronic obstructive pulmonary disease (COPD), and secondary, whether the level of vitamin K is associated with COPD severity, smoking exposure, or mortality. **Methods:** The plasma concentration of dephosphorylated uncarboxylated (dp-uc) MGP was used as an inverse biomarker for vitamin K in 98 COPD patients from the CODEX-P COPD study and 986 controls from the DanFunD study. Low vitamin K status was defined as the upper quartile of dp-ucMGP (>589 pmol/L). Using a logistic regression model, we examined whether low vs. high/moderate vitamin K status increased the odds ratio (OR) of having COPD. Secondary analyses, in the COPD cohort only, examined the association between low vitamin K status and COPD severity, smoking exposure in packyears and all-cause mortality, using a Welch’s *t*-test and log-rank test, respectively. **Results:** Low vitamin K status was associated with increased odds of having COPD, OR 9.7 (95% CI [5.5 to 17.5], *p* < 0.001). We found no associations between low vitamin K and COPD severity (est. −0.03, *p* = 0.7; 95% CI [−0.2 to 0.1]), smoking exposure (*p* = 0.7), or all-cause mortality (*p* = 0.5). **Conclusions:** Low vitamin K status was associated with substantially higher odds of having COPD compared to high/moderate vitamin K status. No association was found between low vitamin K status and COPD severity, smoking exposure, or all-cause mortality. Further studies are needed to determine if vitamin K plays a role in the pathophysiology of COPD and whether supplement therapy is indicated.

## 1. Introduction

Chronic obstructive pulmonary disease (COPD) is a leading cause of morbidity and mortality with 600 million cases expected by 2050. An urgent need for improved preventive and therapeutic strategies such as reliable biomarkers that are able to predict disease progression is of great need [1,2].

Recent studies have highlighted vitamin K as a possible factor in preventing cardiovascular and respiratory diseases [3] by activating the vitamin K-dependent protein Matrix Gla Protein (MGP).

MGP plays a significant role in inhibiting soft-tissue calcification and elastin degradation, which is crucial in preventing lung tissue calcification. Measuring MGP in its inactive form as dephosphorylated uncarboxylated Matrix Gla Protein (dp-ucMGP) can provide insights into the body’s vitamin K status as it serves as an inverse biomarker of vitamin K levels in peripheral tissues [4].

In COPD, vascular calcification and elastin degradation is accelerated contributing to disease pathogenesis. Therefore, it has been hypothesized, through the “Vitamin K deficit and elastolysis theory”, that lower vitamin K, a deficit, could worsen elastin degradation, implying that vitamin K status could be of importance in preventing pulmonary disease progression [5,6].

Emerging research suggests vitamin K may play a role in managing diseases like COPD, though no direct link has been established. Cohort studies have associated low vitamin K with COPD and elastin degradation, indicating its potential importance in pulmonary pathogenesis [7,8]. A large study in the Danish general population found reduced lung function and a higher risk of self-reported COPD in individuals with low vitamin K levels [4]. Furthermore, low vitamin K status has been linked to increased elastin degradation and higher mortality in COPD patients [7]. While these studies have provided important insights, further research not only exploring the role of vitamin K in elastin degradation but also its impact on disease progression, lung function, and long-term survival in larger and diverse patient populations are of need.

This study aims to determine the association between low vitamin K and spirometry-verified COPD compared to a healthy population and test the impact of vitamin K status on COPD severity and mortality.

We hypothesize that individuals with low vitamin K status (upper quartile dp-ucMGP) have higher odds of having COPD compared to a healthy control population and that low vitamin K status is associated with lower lung function and increased all-cause mortality.

## 2. Material and Methods

This is an observational cohort study of a COPD cohort and a healthy control cohort. The COPD cohort derives from the Danish CODEX-P study (“COPD exacerbation and Pulmonary Hypertension”, clinicaltrials.gov: NCT04538976)—a prospective study between 2020 and 2022 comparing transthoracic echocardiographic parameters during hospital-requiring COPD exacerbation and the stable phase. The control group was included from the DanFunD study (“The Danish Study of Functional Disorders”)—a longitudinal population-based study investigating the prevalence of functional disorders or medically unexplained illnesses in the general population. The data was derived from the 5-year follow-up, performed between 2017 and 2020 at the Center for Clinical Research and Prevention and consisted of parameters from both questionnaires and physical examinations [4].

### 2.1. Outcome

The primary outcome is spirometry-verified COPD. Secondary outcomes, only in the COPD cohort, included FEV_1_ as well as all-cause mortality within 3 years.

### 2.2. Study Participants and Sample Size

The COPD cohort consisted of patients with a registered diagnosis of COPD (ICD-10: DJ44), who were active or former smokers and had available vitamin K biomarker data (dp-ucMGP). Participants under 50, those with coagulation disorders or receiving vitamin K antagonists, and those with significant bleeding or surgery within 3 months were excluded.

The control cohort comprised participants who were current or ever smokers with available vitamin K data. Exclusions included those under 50, individuals with self-reported COPD or FEV_1_ < 80%, and those using blood-thinners, to avoid including vitamin K antagonist users.

### 2.3. Measurement of dp-ucMGP Plasma Levels

dp-ucMGP was measured using IDS-iSYS InaKtif MGP ECLIA assay (ImmunoDiagnostic Systems Holdings PLC, East Boldon, UK). Preanalytical sampling and analysis were performed according to manufacturer’s instructions. The intermediary precision was <10%. All dp-ucMGP values below the lower limit of quantitation (300 pmol/L) were set to 299 pmol/L, to determine status with suitable precision and accuracy according to assay instructions [9].

In the control cohort, analysis of dp-ucMGP was performed continuously during data collection when approximately 200 samples had been collected; thus, duration of storage ranged between 0 and 3 months [4]. In the COPD cohort, blood samples were collected at the date of hospitalization due to acute exacerbation of COPD. Analysis of dp-ucMGP was performed in all available samples in April 2023. Average time of −80 °C freezer storage was mean 1.25 years, for which the dp-ucMGP was shown to be stable [10].

### 2.4. Vitamin K Classification

Vitamin K status was categorized using quartile ranges. The upper 4th quartile of dp-ucMGP levels was defined as “Low vitamin K” status, and levels of dp-ucMGP in the 1st, 2nd, or 3rd quartile were defined as “High/moderate vitamin K” status. See Appendix A Table A1 and Table A2 for more details regarding cut-off levels for dp-ucMGP used in the main and secondary analyses.

### 2.5. Lung Function Measurement

Data on spirometry were collected from the patients’ electronic medical records up to three years prior to inclusion in the COPD cohort. If unavailable, spirometry results during the exacerbation were used. Spirometry in the control cohort was performed according to standards of the protocol described by Jespersen, et al. [4].

### 2.6. Statistical Analyses

Descriptive analysis of demographic and clinical characteristics of the study participants was conducted. Categorial variables are presented as counts and percentages, while continuous variables are presented as mean and standard deviation (SD) if normally distributed. Non-normally distributed variables are presented as median and interquartile ranges (IQRs).

### 2.7. Primary Analysis

The complete cohort was divided into two groups: high/moderate vitamin K or low vitamin K—based on quartile levels of dp-ucMGP in pmol/L (see Appendix A Table A1). A logistic regression was performed to investigate a possible association between low vitamin K status and probability of COPD. The exponential of the model parameters produced the odds ratio (OR). The *p*-value and confidence interval (CI) of the given estimates were assessed. The analysis was adjusted for sex, age, and body mass index (BMI). To evaluate whether kidney function would impact the association between vitamin K status and COPD, adjustments for the estimated glomerular filtration rate (eGFR) and creatinine were included as sensitivity analyses.

A visualization of the probability of COPD stratified by vitamin K status was performed using a logistical regression curve.

### 2.8. Secondary Analyses

**Figure A1:**
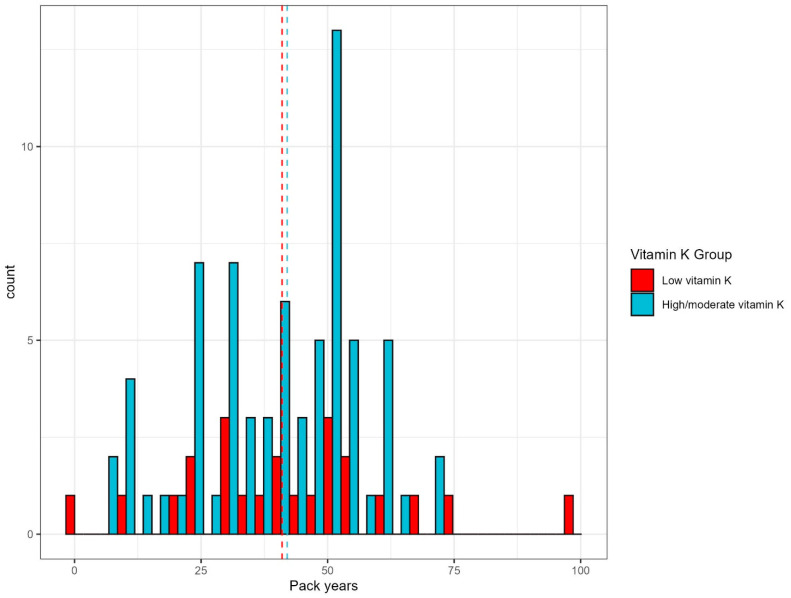
Distribution of pack-years in the COPD-only cohort (CODEX-P) stratified by Vitamin K groups. The dashed lines represent median in pack years for low vitamin K (41) and high/moderate vitamin K (42). One pack-year equals to smoking 20 cigarettes a day for a year. Low vitamin K status is defined as dp-ucMGP levels > 1065.5 pmol/L (upper quartile).

Secondary statistical analyses were performed in only the COPD cohort to examine the association between low vitamin K status and COPD severity, measured as FEV_1_. New dp-ucMGP quartiles were defined only from participants with COPD and were then divided into low or high/moderate vitamin K status (see Appendix A Table A2). A histogram was made to visualize the distribution of lung function in FEV_1_ stratified by vitamin K status. If the groups had same variance, a standard *t*-test was performed—otherwise, a Welch Two-Sample *t*-test was performed. Additionally, the same analyses, a histogram and a Welch Two-Sample *t*-test, were used to determine whether smoking exposure in packyears in the COPD cohort had any significant association with levels of vitamin K (see Appendix A Figure A1). A single packyear was defined as smoking 20 cigarettes a day for a year.

To examine if low vitamin K status and all-cause mortality in the COPD cohort were associated, a Kaplan–Meier curve was fitted. Date of hospital admission was used as start of observation, while the end of observation was set to 30 September 2024. Events was defined as all-cause mortality within this period and were collected from the patients’ electronic medical records. A log-rank test was performed to examine significance.

### 2.9. Data Analysis

All analyses and illustrations were performed using “RStudio”, R version 4.4.1 (14 June 2024 ucrt)—“Race for Your Life”. Copyright © 2024. The R Foundation for Statistical Computing. Platform: ×86_64-w64-mingw32/×64. We considered a *p*-value ≤ 0.05 as statistically significant.

## 3. Results

**Figure 1:**
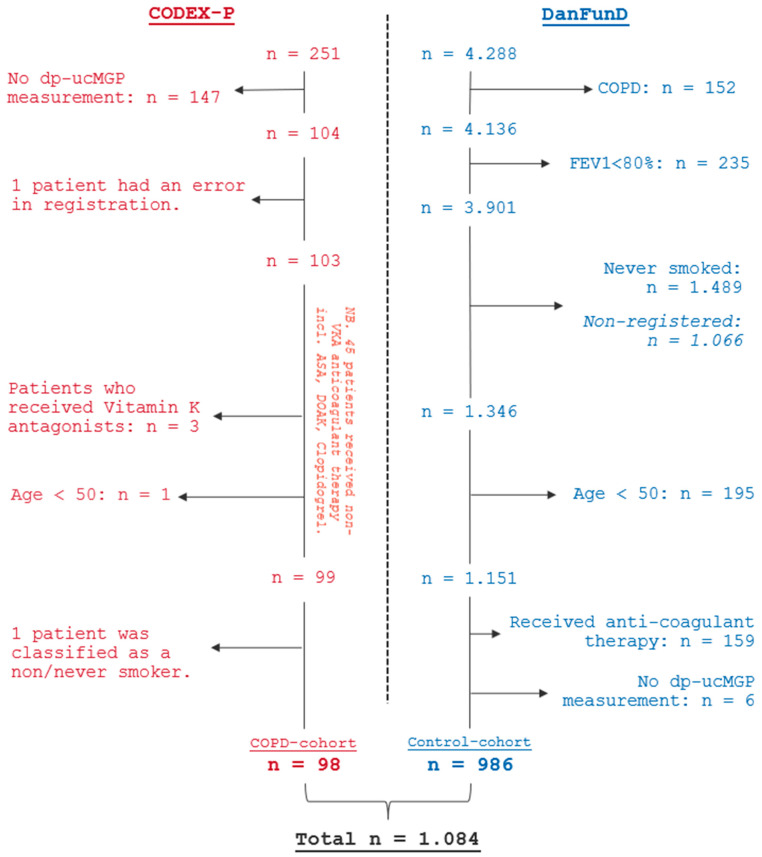
Flowchart—inclusion and exclusion of study participants. Abbreviations: dp-ucMGP, dephosphorylated-uncarboxylated Matrix Gla Protein; FEV_1_, forced expiratory volume in one second; VKA, Vitamin K antagonists; ASA, acetylsalicylic acid; DOAK, direct oral anti-coagulants; NB, Notabene.

The flowchart (Figure 1) visualizes the selection of study participants to the COPD cohort and control cohort. In total, 1084 participants were included: 98 in the COPD cohort and 986 in the control cohort.

The COPD cohort consists of 65.3% female participants, while the control population has 56.9%, with a mean age difference of approximately 10 years (COPD: 74 [8.1], Control: 63 [7.5]). The COPD cohort has lower lung function compared to the control population with a mean (SD) FEV_1_ of 39.96% (16.29) and 101.54% (12.26), respectively. Weekly alcohol use is higher in the COPD cohort (15.87 [17.86] though 84 had missing data) compared to the control population (9.28 [7.92], 32 has missing data), and more participants among the COPD cohort are also active smokers, 33.7% compared to 17.7% in the control population. The majority of participants in the control population have a high/moderate vitamin K status (80.2%), while 19.8% has a low vitamin K status. The median dp-ucMGP level is 485 pmol/L, IQR (409, 570). In the COPD cohort, the median dp-ucMGP level is 734 pmol/L, IQR (591.5, 1.065.5), and 75.5% of participants have a low vitamin K status. (See Table 1)

### 3.1. Odds Ratio of COPD in Low Vitamin K Group

We found an increased odds of having COPD among participants with low vitamin K status compared to those with high/moderate status with an OR of 9.4 (95% CI: 5.4–17.0), *p* < 0.001, (See Table 2) when adjusted for sex, age, and BMI. Two observations were deleted due to missing values of BMI in the COPD cohort. Sensitivity analysis adjusting for creatinine and eGFR separately did not change the association (*p* > 0.05).

**Figure 2:**
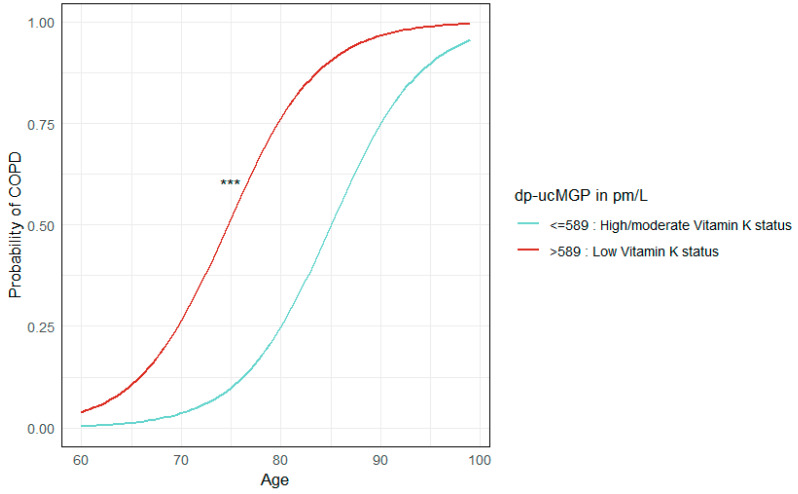
Visualization—risk of COPD in relation to age. Analysis shows probability of COPD in both cohorts (CODEX-P and DanFunD), stratified by Vitamin K status, in relation to age in years. COPD, chronic obstructive pulmonary disease; dp-ucMGP, dephosphorylated-uncarboxylated Matrix Gla Protein. *** refers to *p* ≤ 0.001.

The probability of COPD according to vitamin K status, stratified by age, was visualized using a logistic regression curve (See Figure 2).

### 3.2. Association with Low Vitamin K and COPD Severity and Mortality

**Figure 3:**
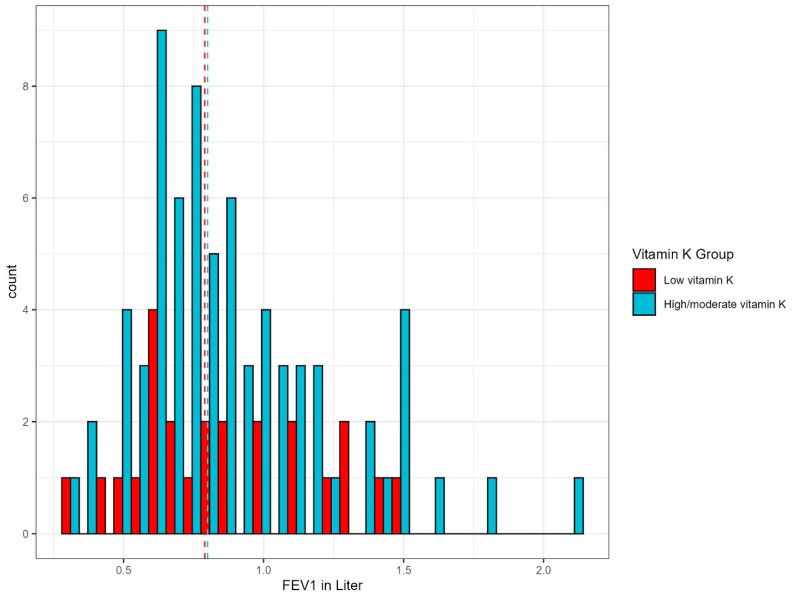
Distribution of FEV_1_ in the COPD only cohort (CODEX-P). FEV_1_; forced expiratory volume in one second. The dashed lines represent the median in FEV_1_ (L) for low vitamin K (0.79 L) and high/moderate vitamin K (0.8 L). Low vitamin K status is defined as dp-ucMGP levels > 1065.5 pmol/L (upper quartile).

Figure 3 visualizes the distribution of lung function measures by FEV_1_ (liter), to approximate COPD severity, stratified by vitamin K status.

A total of 28 participants had no previous lung function measurements in the stable phase prior to inclusion in CODEX-P, and three had no lung function data at all.

There was a difference in variance, with overlapping confidence intervals: the median FEV_1_ for the COPD group with low vitamin K status (dp-ucMGP > 1065.5 pmol/L) was 0.79 L, and 0.8 L for those with high/moderate vitamin levels. Using a Welch Two-Sample *t*-test there was no association between low vitamin K status and the severity of COPD est. −0.03 (*p* = 0.7; CI: [−0.2:0.1]).

Using a Welch Two-Sample *t*-test, we found no significant association between low vitamin K status and smoking exposure measured in pack-years est. 0.50 (*p* = 0.6; CI: [−7.5:12.4]). A visualization of the distribution of smoking exposure measured in packyears stratified by vitamin K status can be found in Appendix A Figure A1.

**Figure 4:**
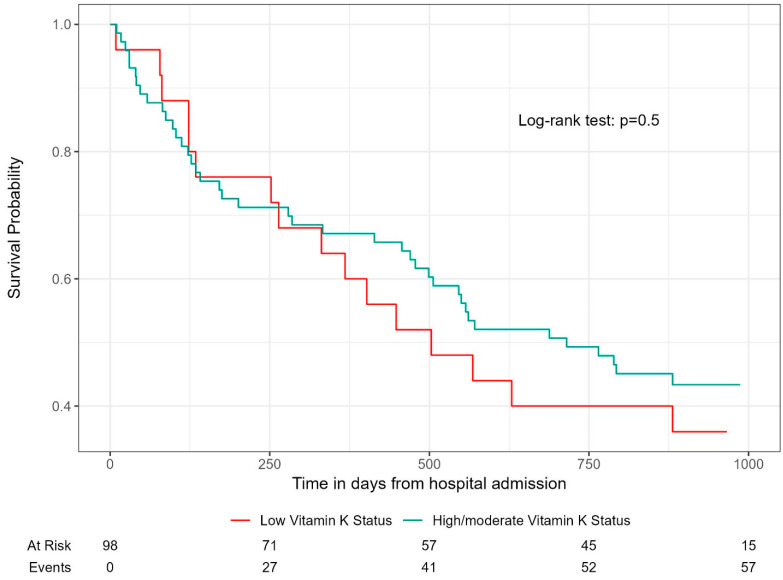
Association between low vitamin K status and mortality in the COPD-only cohort (CODEX-P). Mean time to death is 607 days. Low vitamin K status is defined as dp-ucMGP levels > 1065.5 pmol/L (upper quartile in COPD cohort). Log rank test performed shows insignificant results.

The Kaplan–Meier plot depicting the association between low vitamin K status and all-cause mortality in the COPD cohort showed no statistical difference between low and high/moderate vitamin K status and all-cause mortality (log-rank test *p* = 0.5). See Figure 4.

Since there was no violation of proportional hazards when using cox regression, *p* = 0.6, a standard log rank test was deemed sufficient. After the end of observation (30 September 2024), 57 patients were deceased, while 15 were still at risk. The mean time to death was 607 days.

## 4. Discussion

This study found that low vitamin K status was associated with higher odds of having COPD compared to high/moderate vitamin K status. However, no significant association was found between low vitamin K status and COPD severity or all-cause mortality.

These findings align with previous studies [4,7], although Jespersen et al. found an association between low vitamin K status and reduced ventilatory capacity in the general population, which differs from our findings using spirometry-verified COPD [4]. Our findings correlate with those from Piscaer et al. who found an association between vitamin K status and COPD, and even proposed a biological mechanism of the association in the form of increased elastin degradation due to vitamin K deficiency [7,11].

Regarding the potential directionality of the association between low vitamin K status and COPD, our study suggests that low vitamin K may be a contributing factor to the development of COPD rather than a consequence of the disease itself. The higher odds of COPD in individuals with low vitamin K status and the significantly greater proportion of low vitamin K status among COPD patients support this hypothesis. However, as our study is observational, we acknowledge that interventional studies are needed to fully determine whether low vitamin K status is a predisposing factor for COPD or whether COPD leads to vitamin K deficiency.

We found no associations between vitamin K status and COPD severity, consistent with previous findings [7]. However, we did not collect data on diffusing capacity for carbon monoxide nor data on the degree of emphysema, which may provide valuable insights into the association between vitamin K status and COPD severity. It is important to note that, once COPD is established, other factors such as airway inflammation, oxidative stress, and structural lung changes (e.g., emphysema) may become more influential in disease progression. It is possible that the effects of vitamin K on elastin degradation and vascular health are more prominent in the early stages of the disease, whereas, in more advanced stages, the overall disease burden may overshadow the impact of vitamin K status on disease severity.

We also observed no difference in mortality, which is in contrast with findings by Piscaer et al. who found an association between low vitamin K and 5-year mortality [7]. We cannot exclude that longer observation time in our study would have resulted in an increased mortality risk. Further, our study was a post-analysis and not powered for the secondary analyses.

Strengths of our study include the use of a well-defined cohort with spirometry-verified COPD, providing reliable data on disease presence and severity. Additionally, vitamin K status was objectively measured using dp-ucMGP, ensuring precise biomarker data. Finally, this study’s combination of a healthy control group and a large COPD cohort allows for an evaluation of the role of vitamin K in disease development and progression in a realistic clinical context.

Our study specifically examines low vitamin K status and the odds of having COPD in a large control cohort of healthy individuals and a case–cohort diagnosed with severe spirometry-verified COPD. Though we found similar results concordant with previous studies [4,7], following limitations, interactions and possible confounding effects need to be addressed.

First, vitamin K is largely dependent on diet, and individuals with malabsorption disorders are at higher risk of deficiency [12]. Around one in three inpatients and one in five outpatients with COPD are at risk of malnutrition, which worsens after exacerbations [13,14]. However, in the COPD cohort, nearly 75% had low vitamin K status, yet none had known gastrointestinal disorders. Second, the COPD cohort’s average alcohol intake was higher than controls. Excessive alcohol can lead to vitamin K deficiency by disrupting nutrient absorption, potentially affecting our findings [15,16,17,18]. Unfortunately, we were not able to adjust for alcohol intake due to missing data. Third, all COPD participants were hospitalized due to AECOPD and received antibiotics, before vitamin K assessment. Critical illness and antibiotics use may lead to vitamin K deficiency by disrupting gut bacteria and affecting nutrient absorption. These factors, along with inadequate diet and increased metabolic demands, may have influenced vitamin K uptake [19]. Fourth, samples from the COPD cohort had been stored for about a year longer than controls, which may have led to the underestimation of vitamin K differences due to potential degradation.

### Perspective

Vitamin K measurements in COPD patients are currently not of use clinically, but confirming their diagnostic value and role in risk assessment may be beneficial. Randomized trials on vitamin K supplementation could reveal its impact on COPD management, enabling tailored treatments or aiding early detection in high-risk individuals.

## 5. Conclusions

In this observational study, we found a marked increase in odds of having COPD in participants with a low vitamin K status. No association was found between low vitamin K status and COPD severity, as measured by FEV_1_, or all-cause mortality.

Our results indicate that vitamin K may be of importance as a biomarker of COPD in high-risk patients. Clinical trials are needed to establish a clear causal relationship and to confirm the potential therapeutic role of vitamin K in COPD, especially in reducing pulmonary calcification, inflammation, or improving overall outcomes.

## Figures and Tables

**Table 1:** Participant characteristics.

	COPD	Control
*n*	98	986
Sex, Female (%)	64 (65.3)	561 (56.9)
Age, years (mean (SD))	74.9 (8.1)	63.1 (7.5)
Height, cm (mean (SD))	165.8 (8.7)	170.9 (9.1)
Weight, kg (mean (SD))	67.5 (18.6)	77.3 (14.9)
BMI, kg/m^2^ (mean (SD))	24.5 (6.1)	26.4 (4.1)
Diabetes type 1/2 (%)	13 (13.3)	40 (4.1)
Hypertension (%)	49 (50.5)	348 (35.3)
Systolic BP (mean (SD))	139.2 (22.6)	132.7 (18.5)
Diastolic BP (mean (SD))	80 (15.9)	80.6 (10.3)
Asthma (%)	17 (17.3)	87 (8.8)
FEV_1_ L (mean (SD))	0.88 (0.34)	2.81 (0.64)
FEV_1_ % (mean (SD))	39.96 (16.29)	101.54 (12.26)
FVC liter (mean (SD))	1.90 (0.61)	3.82 (0.88)
FVC % (mean (SD))	69.14 (23.74)	112.48 (13.64)
FEV_1_/FVC ratio (mean (SD))	0.47 (0.14)	0.74 (0.08)
Creatinine, µmol/L (median [IQR])	69 [55, 86.5]	74 [66, 84.8]
eGFR, mL/min/1.73 m^2^ (median [IQR])	81.2 [64.4, 92.2]	83.4 [73.5, 91.2]
Alcohol use weekly, units (mean (SD))	15.9 (17.9) Missing: 83	9.3 (7.9) Missing: 32
Smoking status (%)		
Current	33 (33.7)	175 (17.7)
Ever smoker	65 (66.3)	811 (82.3)
Vitamin K status (%)		
* Low	74 (75.5)	195 (19.8)
** High/moderate	24 (24.5)	791 (80.2)
dp-ucMGP, pmol/L (median [IQR])	743 [591.5, 1.065.5]	485 [409, 570]

**Abbreviations:** BMI, body mass index; BP, blood pressure; FEV_1_, forced expiratory volume in one second; FVC, forced vital capacity; eGFR, estimated glomerular filtration rate; and dp-ucMGP, dephosphorylated-uncarboxylated Matrix Gla Protein. * Low: Refers to levels of dp-ucMGP >589 pmol/L. ** High/moderate: Refers to levels of dp-ucMGP between 299 and 589 pmol/L.

**Table 2:** Risk of COPD stratified by vitamin K status.

Coefficients	Estimates (SD)	OR	2.5%	97.5%	*p* Value
* High/moderate vitamin K	ref	ref	ref	ref	ref
** Low vitamin K	2.27 (0.29)	9.7	5.5	17.5	<0.001
Sex (male)	−0.24 (0.29)	0.8	0.4	1.4	0.409
Age	0.22 (0.03)	1.25	1.2	1.3	<0.001
BMI	−0.13 (0.03)	0.9	0.8	0.9	<0.001

Analysis included both cohorts (CODEX-P and DanFunD) and was adjusted for sex, age, and body mass index (BMI). OR: odds ratio, SD: standard deviation. Ref: reference * High/moderate vitamin K status was determined to be levels of dp-ucMGP between 299 and 589 pmol/L. ** Low vitamin K status was determined to be levels of dp-ucMGP > 589 pm/L.

## Data Availability

It is the opinion of Copenhagen Respiratory Research (COP:RESP) that knowledge sharing leads to more and better scientific outcomes. Requests for trial information can be submitted to the Project Management team (Jens-Ulrik Jensen, Pradeesh Sivapalan), who will review the request. Any reasonable requests will then be discussed with the CODEX-P research group. If the research group agrees to share the data, approval must first be obtained from the Danish authorities. Restrictions apply to the availability of DanFunD data according to Danish law to protect sensitive participant information. Requests for access to data need approval from appropriate Danish authorities as the data are subject to Danish regulations on personal data protection, which also encompass requirement for long term storage and data availability. A request for the approval and arrangement of DanFunD data transfer agreements can be sent to Center for Clinical Research and Prevention, who host the data; ckff@regionh.dk.

## Appendix A

**Table A1:** Quartile ranges of dp-ucMGP in main analysis—all participants.

Quartile ranges ALL	Min.		25%		Median		75%		Max.
Levels of dp-ucMGP in pmol/L	299	-	411.75	-	495.5	-	589	-	3114
Vitamin K Group	High/moderate vitamin K						Low vitamin K		

**Table A2:** Quartile ranges of dp-ucMGP in secondary analyses—COPD-only cohort (CODEX-P).

Quartile ranges COPD	Min.		25%		Median		75%		Max.
Levels of dp-ucMGP in pmol/L	299	-	591.5	-	734	-	1065.5	-	3114
Vitamin K Group	High/moderate vitamin K						Low vitamin K		
